# Sporadic Noradrenergic Adrenal Pheochromocytoma in an Adolescent Patient

**DOI:** 10.7759/cureus.19443

**Published:** 2021-11-10

**Authors:** Sasi K Penukonda, Craig B Chu

**Affiliations:** 1 Pediatric Endocrinology, Willis-Knighton Health System, Shreveport, USA; 2 Pediatrics, Willis-Knighton Health System, Shreveport, USA

**Keywords:** adrenal, metanephrines, catecholamines, paraganglioma, pheochromocytoma

## Abstract

Pheochromocytoma and paraganglioma are neuroendocrine tumors that occur less commonly among children compared to adults. The excess catecholamines secreted by the tumor cells result in hypertension, tachycardia, excess sweating, and headache. Other symptoms include abdominal pain or distension caused by the adrenal mass. Here, we report a case of pheochromocytoma arising from the left adrenal medulla in a 14-year-old boy, which was exclusively secreting norepinephrine, as suggested by elevated plasma and 24-hour urinary norepinephrine and its metabolite normetanephrine. The epinephrine and its metabolite metanephrine were within normal limits. He presented with abdominal pain, recurrent vomiting, and headache and was noted to have elevated blood pressure. He underwent adrenalectomy after controlling his blood pressure with an alpha-blocker Prazosin. His blood pressure remained stable after surgery, and his plasma-free metanephrines returned to normal limits. He tested negative for hereditary paraganglioma-pheochromocytoma gene panel.

## Introduction

Pheochromocytoma and paraganglioma are rare neuroendocrine tumors arising from the catecholamine-producing chromaffin cells of the adrenal medulla or paraganglia with an estimated incidence of 0.11 per million children [1]. Hypertension is the most consistent manifestation of these tumors, and it is more likely seen in children with or without paroxysmal crisis superimposed [2]. The excess catecholamines produced are mostly metabolized to metanephrines within the tumor cells by membrane-bound catecholamine O-methyltransferase [3]. Hence, the Endocrine Society guidelines recommend the measurement of plasma-free metanephrines or urinary fractionated metanephrines to diagnose pheochromocytoma and paraganglioma [4]. The catecholamine phenotype varies in pheochromocytoma and paraganglioma, with most paragangliomas presenting with lower epinephrine and metanephrine plasma concentrations compared to pheochromocytoma [5]. Here, we report a case of pheochromocytoma in a 14-year-old adolescent male patient with excess norepinephrine and normetanephrine but normal epinephrine and metanephrine levels.

## Case presentation

**Figure 1:**
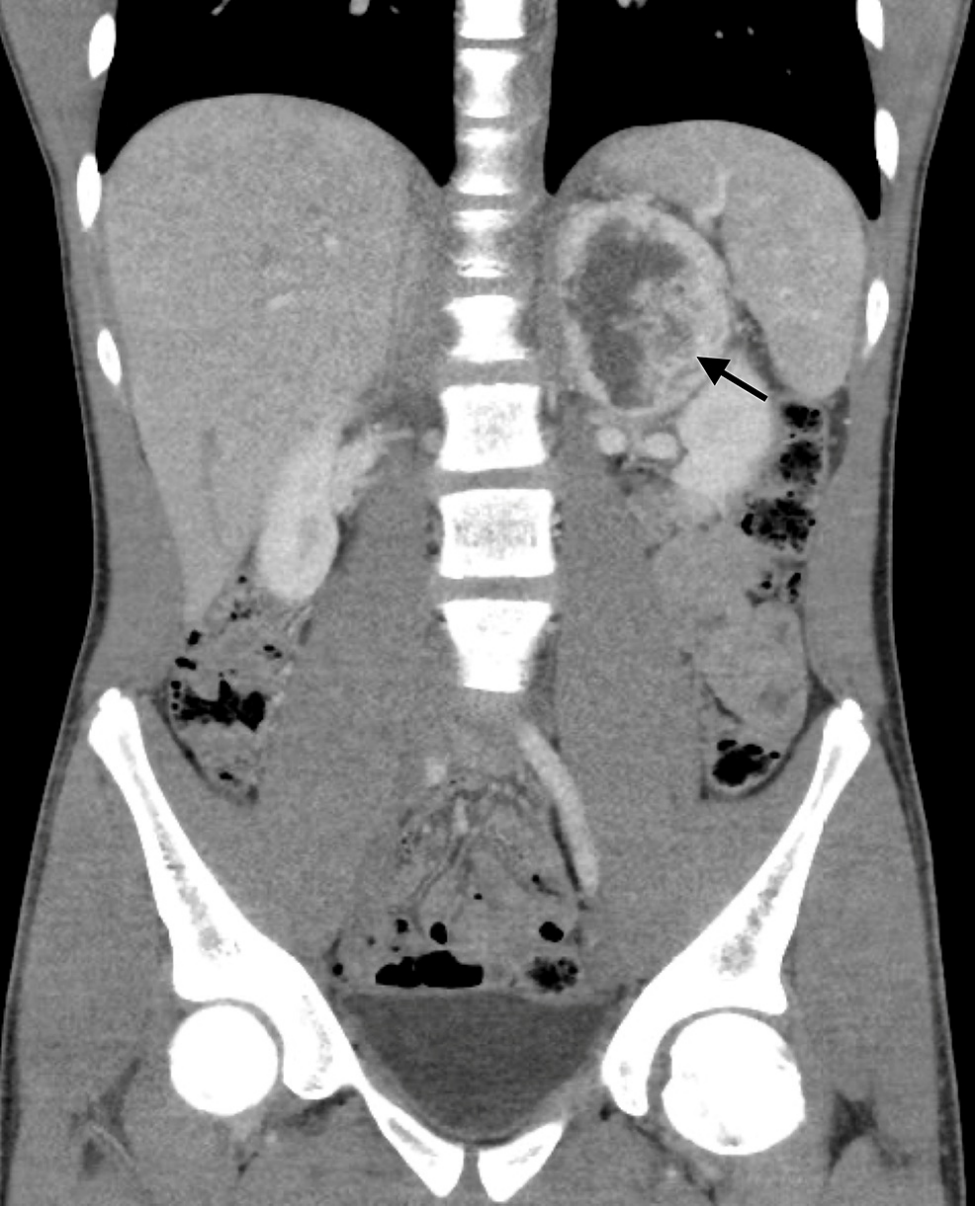
Coronal MPR CT image with contrast demonstrating heterogeneously enhancing left adrenal mass (black arrow). MPR: multiplanar reformation; CT: computed tomography

A 14-year-old adolescent male patient presented with incidentally discovered left adrenal mass measuring 6 × 6 × 7.4 cm on a computed tomography (CT) scan (Figure 1) while being investigated for vague left upper abdominal pain and recurrent vomiting. On further questioning, he reported frequent headaches. There was no history of palpitations or excess sweating. His blood pressure was elevated around 140/78 mmHg, and his heart rate was 103 beats per minute.

Further investigations revealed elevated plasma-free normetanephrine and 24-hour urine normetanephrine and norepinephrine. Plasma-free metanephrine and 24-hour urine metanephrine and epinephrine were within normal limits (Table 1).

**Table 1:** Laboratory findings at initial presentation.

Plasma-free metanephrines		24-hour urinary fractionated metanephrines		24-hour urinary fractionated catecholamines	
Free normetanephrine	Free metanephrine	Normetanephrine	Metanephrine	Norepinephrine	Epinephrine
30 nmol/L	<0.2 nmol/L	8,887 µg/24 hour	92 µg/24 hour	574 µg/24 hour	1.9 µg/24 hour
Reference range					
<0.9 nmol/L	<0.5 nmol/L	91–456 µg/24 hour	69–221 µg/24 hour	15–80 µg/24 hour	0.5–20 µg/24 hour

**Figure 2:**
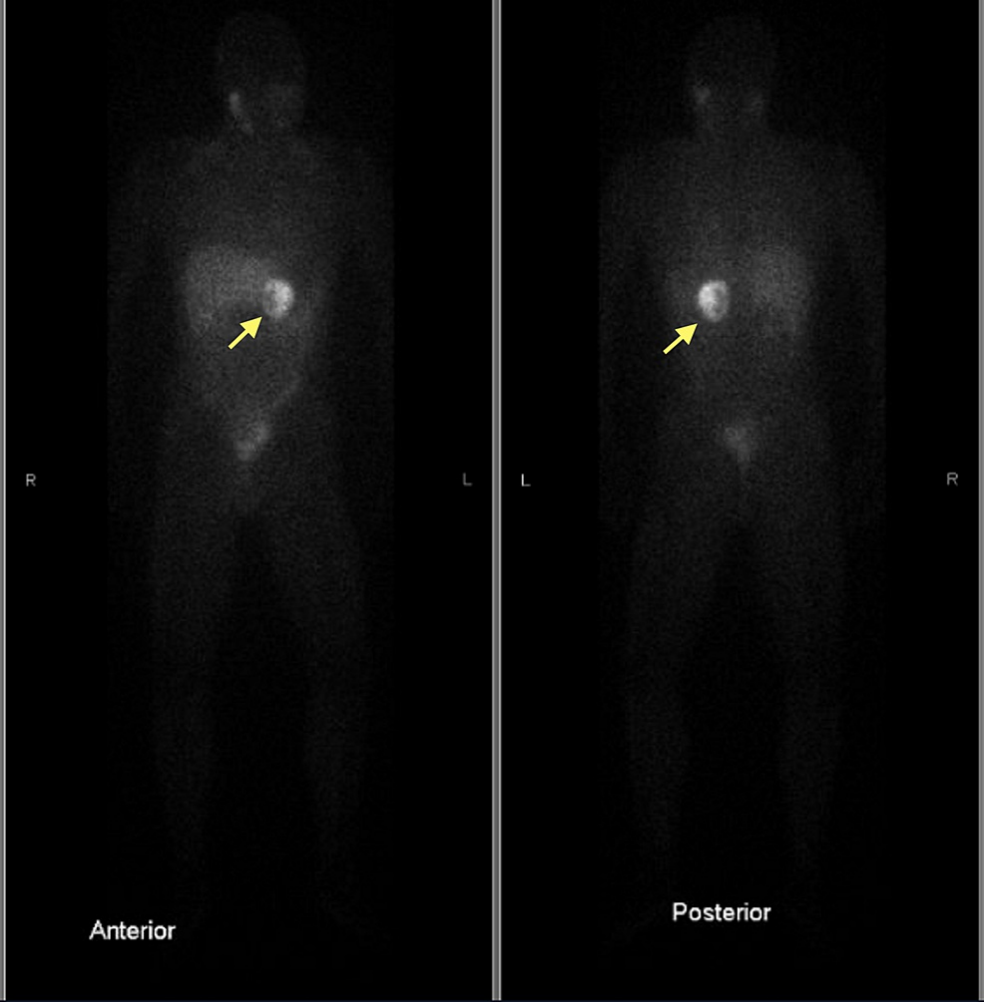
MIBG scan images showing intense uptake of iodine-123 in the region of the left adrenal mass (yellow arrow). MIBG: meta-iodobenzylguanidine

Iodine-123 meta-iodobenzylguanidine scan revealed intense uptake in the region of mass in the left adrenal gland. No other abnormal areas of uptake were noted throughout the chest, abdomen, pelvis, extremities, head, and neck (Figure 2).

He was treated with an alpha-blocker prazosin for two weeks prior to adrenalectomy to control his blood pressure. He was also advised a high-salt diet and increased fluid intake to expand blood volume and prevent hypotension after removal of the tumor. The patient underwent robot-assisted left adrenalectomy, and his postoperative period was uneventful. Prazosin was discontinued as his blood pressure remained stable after surgery. Biopsy confirmed the diagnosis of pheochromocytoma. His plasma-free metanephrine levels two weeks after surgery were normal (Table 2).

**Table 2:** Plasma-free metanephrine levels before and after surgery.

Plasma-free metanephrines	Before surgery	Two weeks after surgery	Reference range
Free normetanephrine	30 nmol/L	0.59 nmol/L	<0.9 nmol/L
Free metanephrine	<0.2 nmol/L	<0.2 nmol/L	<0.5 nmol/L

He tested negative for hereditary paraganglioma-pheochromocytoma gene panel, including *Von Hippel-Lindau* (*VHL*), *succinate dehydrogenase complex subunit B* (*SDHB*), *succinate dehydrogenase complex subunit D* (*SDHD*), and *RET *genes.

## Discussion

Pheochromocytomas and paragangliomas are heterogenous tumors with diverse phenotypes. Paragangliomas predominantly or exclusively secrete norepinephrine, whereas epinephrine secretion is usually confined to pheochromocytomas arising from the adrenal medulla [6]. The excess production of epinephrine limited to tumors arising from the adrenal medulla is thought to be due to the proximity of these tumors to adrenal cortical steroids, which induce the production of phenylethanolamine-N-methyltransferase (PNMT), the enzyme that converts norepinephrine to epinephrine [7]. However, it was later demonstrated that neither in vivo (direct contact between pheochromocytes and cortical cells) nor in vitro (pheochromocytes treated with dexamethasone) is sufficient to ensure PNMT transcription [5].

Approximately one-half of adrenal tumors produce nearly exclusively norepinephrine, and the other half produce a variable mixture of epinephrine and norepinephrine [8,9]. Our patient had adrenal pheochromocytoma exclusively producing norepinephrine. One study revealed that noradrenergic tumors usually present with sustained hypertension, as seen in our patient, and the tumors producing high levels of epinephrine cause paroxysmal hypertension [10]. More than 50% of patients aged less than 20 who present with pheochromocytoma/paraganglioma were found to have identifiable germline mutations, and the most common genes involved were *VHL*, *RET*, *SDHD*, and *SDHB* [11]. The catecholamine phenotype in hereditary pheochromocytomas can vary with underlying gene mutation, with those from Von Hippel-Lindau syndrome (*VHL *gene mutation) producing predominantly norepinephrine, and the tumors from multiple endocrine neoplasia type 2 (*RET* gene mutation) producing a mixture of epinephrine and norepinephrine [12]. Our patient tested negative for all common genetic mutations associated with pheochromocytoma, suggesting sporadic type.

Surgical excision is the treatment of choice. Hormonally functional pheochromocytomas need treatment with alpha-blockers such as phenoxybenzamine or prazosin to prevent perioperative cardiovascular complications. Another option is the administration of calcium channel blockers. Beta-blockers can be added to control tachycardia after administration of alpha-blockers. Other supportive care includes a high-sodium diet and increased fluid intake to reverse catecholamine-induced blood volume contraction preoperatively, and thereby prevent severe hypotension after tumor removal [4,13].

Recent advances in laparoscopic surgery have increased the feasibility of laparoscopic adrenalectomy in pheochromocytoma [14,15]. Open surgery is recommended for tumors greater than 6 cm as it allows complete removal of the tumor and minimizes systemic catecholamine release [4]. Adrenal “cortical sparing” procedures have been advocated for patients with bilateral tumors to avoid the need for long-term steroid replacement and the risk of Addisonian crisis [15]. Our patient underwent robot-assisted adrenalectomy with one of the abdominal incisions widened to remove the intact adrenal mass.

## Conclusions

Pheochromocytoma and paraganglioma are relatively rare in the pediatric population. Children presenting with adrenal mass or hypertension should be investigated for pheochromocytoma. Measurement of catecholamine metabolites such as normetanephrine and metanephrine in plasma and 24-hour urine helps accurately diagnose these tumors. Children diagnosed with pheochromocytoma/paraganglioma need further evaluation to rule out germline mutations associated with these tumors.
